# Dispersal of human and plant pathogens biofilms via nitric oxide donors at 4 °C

**DOI:** 10.1186/s13568-016-0220-1

**Published:** 2016-07-26

**Authors:** Massimiliano Marvasi, Ian A. Durie, Tania Henríquez, Aiste Satkute, Marta Matuszewska, Raphael Carvalho Prado

**Affiliations:** 1 Department of Natural Sciences, School of Science and Technology, Middlesex University, The Burroughs, London, NW4 4BT UK; 2 Soil and Water Science Department, University of Florida, Gainesville, FL USA; 3 Department of Microbiology and Mycology, Institute of Biomedical Sciences (ICBM), University of Chile, Santiago, Chile

**Keywords:** *Salmonella enterica*, Biofilms, Nitric oxide donors, MAHMA NONOate, Biofilm dispersal, Sanitization

## Abstract

Recent studies suggest that nitric oxide donors capable of manipulating nitric oxide-mediated signaling in bacteria could induce dispersal of biofilms. Encased in extracellular polymeric substances, human and plant pathogens within biofilms are significantly more resistant to sanitizers. This is particularly a problem in refrigerated environments where food is processed. In an exercise aimed to study the potential of nitric oxide donors as biofilm dispersal in refrigerated conditions, we compared the ability of different nitric oxide donors (SNAP, NO-aspirin and Noc-5) to dislodge biofilms formed by foodborne, human and plant pathogens treated at 4 °C. The donors SNAP and Noc-5 were efficient in dispersing biofilms formed by *Salmonella enterica*, pathogenic *Escherichia coli* and *Listeria innocua*. The biomasses were decreased up to 30 % when compared with the untreated controls. When the plant pathogens *Pectobacterium* sp. and *Xanthomonas* sp. were tested the dispersion was mainly limited to *Pectobacterium carotovorum* biofilms, decreasing up to 15 % after exposure to molsidomine. Finally, the association of selected nitric oxide donors with sanitizers (DiQuat, H_2_O_2_, peracetic acid and PhenoTek II) was effective in dispersing biofilms. The best dispersal was achieved by pre-treating *P. carotovorum* with molsidomine and then peracetic acid. The synergistic effect was estimated up to ~35 % in dispersal when compared with peracetic acid alone. The association of nitric oxide donors with sanitizers could provide a foundation for an improved sanitization procedure for cleaning refrigerate environments.

**Electronic supplementary material:**

The online version of this article (doi:10.1186/s13568-016-0220-1) contains supplementary material, which is available to authorized users.

## Introduction

Nitric oxide has recently attracted attention due to its potentiality as signaling molecule and for several biotechnological applications (Moncada et al. 1991; Gasco et al. 1996; Chen et al. 2013). Nitric oxide is currently used in medicine mediating vasodilation, and it has recently showed a great potential as a molecule able to dislodge biofilms (Wang et al. 2005; Barraud et al. 2006). During biofilm dispersal, nitric oxide works as a messenger rather than a generic poison (Barraud et al. 2006; Barraud et al. 2009a, b). It can be delivered as a gas or via donor molecules (Wang et al. 2005; Barraud et al. 2009b) and the nitric oxide releasing rate is mediated by the chemical structure of the donor itself (Wang et al. 2005). Donors release nitric oxide in different ways: pH-dependent manner, via enzymatic reactions, photo or temperature sensitive manner (Maragos et al. 1991; Keefer et al. 1996; Wang et al. 2005).

In bacteria, nitric oxide seems to have a dual effect: it reduces bacterial adhesion (Charville et al. 2008) and promotes biofilm dispersal (Barraud et al. 2009a, b; Marvasi et al. 2014, 2015). Pioneering studies by Barraud and co-workers (2006) firstly showed the potential dispersion of biofilm preformed by *Pseudomonas aeruginosa*. Dispersal was induced with low, sub-lethal concentrations (25–500 nmol/L) of the nitric oxide donor sodium nitroprusside (SNP) (Barraud et al. 2006). Other studies showed the dispersal potential of donors such as molsidomine, MAHMA NONOate, diethylamine NONOate diethylammonium, PROLI NONOate (Marvasi et al. 2014; Barnes et al. 2015). The mechanisms leading to the NO donor-mediated dispersal of biofilm are not completely clear, but it appears to function in the transition of sessile biofilm organisms to free-swimming bacteria (Barraud et al. 2015). Genetic studies have revealed that genes involved in nitric oxide signaling are regulated in both Gram-positive and Gram-negative bacteria showing a universal regulation of nitric oxide in bacteria (Firoved et al. 2004; Xiong and Liu 2010; Narayanasamy 2011; Hong et al. 2014). Biofilms can form recalcitrant reservoirs of bacteria that affect water quality, leading to diseases and post-harvest losses. It is clear that an effective dispersal and removal of these biofilms can benefit the food industry.

Microbes within biofilms are encased in various polymers and are significantly more resistant to sanitizers (Corcoran et al. 2014). The association of nitric oxide donor(s) with sanitizers or detergents treatments was suggested as a hurdle technology to improve the effectiveness of sanitization (Barraud et al. 2006). The dispersal of bacteria with nitric oxide donors coupled with the sanitizers treatment could have a synergistic effect: While nitric oxide induces the transition from biofilm to planktonic state, the sanitizer can easily kill free-swimming cells. Only limited literature is available on the association of sanitizers and donors. Synergistic effects of H_2_O_2_, the industrial sanitizer SaniDate 12.0 and the cellulose hydrogel nanocrystal (CNC) in dispersing *P. aeruginosa*, *Salmonella* and *Escherichia coli* were reported at 22 °C. The synergistic effect of 500 nM sodium nitroprusside with 1 ppm H_2_O_2_ was very effective; Log 2.5 CFU/cm^2^ of reduction of *P. aeruginosa* of CFU recovered from treated surfaces was measured. In the other two cases, the association of SaniDate 12.0 with 10 nM molsidomine and MAHMA NONOate increase the dispersal of *Salmonella* biofilms by 20 % when compared with the sanitizer alone. With reference to the synergistic effect of CNC with 1 mM MAHAMA NONOate, the association of the two molecules was able to disperse 1 week-old *Salmonella* biofilm, otherwise impossible with the sole use of the donor (Barraud et al. 2009b; Marvasi et al. 2014, 2015).

The effectiveness of nitric oxide donor has been mainly studied at room temperature (about 22 °C) and only minor evidences show biofilm dispersal at 4 °C (Marvasi et al. 2014). The advantage to sanitizers cold rooms without to shot down the system is evident: It saves money, time and it is the preferential approach in large cold walk-in environments.

Our aim is to measure to what extent the efficacy of selected nitric oxide donors can be used in refrigerated conditions in association with sanitizers. The implications of this observation for industrial applications are interesting: The ability of the nitric oxide donors to disperse biofilms at 4 °C makes them good candidates for cleaning refrigerated surfaces, common in the food industry.

## Materials and methods

### Bacterial strains and culture media

The pathogenic *E. coli* strains were isolated from outbreaks attributed to vegetables: *E. coli* O157:H7 LJH0537, *E. coli* O157:H7 LJH1186, *E. coli* O157:H7 LJH643, *E. coli* O145 RM12333 (Selma et al. 2008). *Salmonella enterica* (isolated from vegetables outbreaks): *S. enterica* serovar Typhimurium ATCC14028, sv. Braenderup 04E01347, Braenderup 04E01556, Braenderup 04E00783, sv. Montevideo LJH519, sv. Javiana ATCC BAA-1593 and sv. Newport C6.3 (Noel et al. 2010). *Listeria innocua* ATCC33090 was purchased from ATCC (Teddington, Middlesex, UK). We were also interested in testing the effect of nitric oxide donors on dispersing biofilm formed by plant pathogens; It is well know that they can form biofilm in irrigation pipes (Narayanasamy 2011; Hong et al. 2014). The following plant pathogens were used: *Pectobacterium carotovorum* SR38, and *Xanthomonas oryzae pv.oryzae* J18. All strains were maintained as frozen glycerol stocks, and were sub-cultured into Luria–Bertani (Fisher, Waltham, MA, USA), Nutrient Agar (Oxoid, Basingstoke UK) or Brain Heart Infusion broth (Oxoid, Basingstoke UK) media.

### Nitric oxide donors

The following criteria were used to select candidate nitric oxide donors: (1) low/moderate toxicity; (2) no more than 0.1 % of probable, possible or confirmed human carcinogenicity according to the International Agency for Research on Cancer (IARC); (3) low/moderate cost; (4) commercial availability.

Nitric oxide donors used in this study: *S*-nitroso-*N*-acetyl-d,l-penicillamine (SNAP) (Cayman Chemicals, Ann Arbor, MN, USA), 3-(aminopropyl)-1-hydroxy-3-isopropyl-2-oxo-1-triazene (Noc-5), 2-(acetyloxy)benzoic acid 4-(nitroxymethyl)phenyl ester (NO-aspirin), 6-(2-hydroxy-1-methyl-2-nitrosohydrazino)-*N*-methyl-1-hexanamine (MAHMA NONOate), and molsidomine (all from Sigma-Aldrich, St. Louis, MO, USA). For each compound, 1 mM stock solutions were prepared in phosphate-buffered saline, pH 7.3 (PBS, Fisher, Waltham, MA, USA) and aliquots were stored at −80 °C. For the assays, serial dilutions were always prepared fresh in PBS just before the experiments and used within 5 min of their preparation. Biofilm dispersion potential of the molecules was tested on polystyrene and polypropylene 96 well-plates (Fisher, Waltham, MA, USA).

### Biofilm formation and dispersal on plastics

Overnight cultures (10^8^ CFU/mL) grown in appropriate media were diluted in 1:100 of the following media: in colony-forming antigen (CFA) (Teplitski et al. 2006) broth medium for *Salmonella and E. coli*, Nutrient Agar for *Pectobacterium carotovorum* SR38 (bacterial soft rot), and *Xanthomonas campestris* J18 (bacterial spot). For *L. innocua* Brain Heart Infusion broth with 1 % glucose (Fisher, Waltham, MA, USA) was used (Marvasi et al. 2014). Hundred microlitre of the diluted cultures were aliquoted into wells of 96-well polypropylene or polystyrene plates (Fisher, Waltham, MA, USA). Plates with bacteria were incubated for 18 h at 37 °C for *Salmonella*, *E. coli*, *L. innocua* and 48 h at 30 °C for *P. carotovorum* SR38, *X. oryzae pv.oryzae* J18 inside a Ziploc bag to prevent evaporation. Biofilms were formed in the dark in static incubation. Upon completion of the incubation, medium was removed by aspiration and 200 µL aliquots of serial dilutions of nitric oxide donors in PBS were added to the wells with biofilms. Dispersal experiments were conducted at 4 °C for 6 h. Dispersal was measured by staining the remaining biofilms with 1 % (w/v) crystal violet in ethanol and de-staining with acetic acid 33 % (v/v), as described previously (O’Toole and Kolter 1998; Merritt et al. 2005). Three biological and four technical replicates for each experiment were tested. Percentage of dispersal was calculated by dividing the optical density of the treated by the control optical density. The result was multiplied by hundred. When cocktails strains were used, 10^8^ cell/mL from each strain were mixed in the same proportion before biofilm formation.

### Additive effect of the sanitizers with nitric oxide donors

Biofilms of *P. carotovorum* SR38*, S. enterica* sv Typhimurium ATCC14028, and *L. innocua* were set up as above using overnight cultures of the pathogen diluted 1:100 in the CFA or Nutrient Agar medium, where appropriate, in wells of 96-well polypropylene plates (Fisher, Waltham, MA, USA). Plates with bacteria were incubated as above inside a Ziploc bag. Upon completion of the incubation, the medium with planktonic bacteria was removed by aspiration and 200 µL aliquots of serial dilutions of nitric oxide donors in PBS were added to the biofilms. As control, PBS alone was used. Plates were incubated at 4 °C for 6 h. Upon completion of the incubation, planktonic cells were removed by aspiration, wells were washed with PBS and 200 µL of the following sanitizers, diluted as per manufacturer’s recommendations, were loaded into the wells: H_2_O_2_ (final concentration 2 % v/v), peracetic acid (10 % v/v) (Sigma-Aldrich, St. Louis, MO, USA), quaternary ammonium compound Diquat (500 mg/L) (Nufarm, Morrisville, NC, USA) or Pheno-Tek II (0.3 % w/v) (Bio-Tek Industries, Atlanta, GA, USA). The biofilms were incubated with sanitizers for 10 min at 4 °C, after which biofilm dispersal was measured by staining the remaining biofilms with 1 % crystal violet in ethanol, as described previously (O’Toole and Kolter 1998; Merritt et al. 2005). Three biological and four technical replicates for each experiment were tested.

### qPCR to verify the expression of nitric oxide related genes in Salmonella

Five millilitre of planktonic cells exposed at 22 °C to 1 nM donor MAHMA NONOate for 45 min or PBS (as control) were recollected. Total RNA was extracted from samples using mirVana™ miRNA Isolation Kit (Life Technologies) according to the manufacturers’ instructions. RNA integrity was visualized on 1.3 % agarose gel electrophoresis. Samples were quantified with Nanodrop Spectrophotometer (ThermoFisher Scientific) according to manufacturers’ instructions. DNA was removed with TURBO DNA-freeTM Kit (Life Technologies). cDNA synthesis was performed by using Transcriptor First Strand cDNA Synthesis Kit (Roche) according with the user manual by using random hexamer primers. qPCR was performed on a qPCR LightCycler 96 System (Life Technologies) instrument by using PCRBIO SyGreen Mix Hi-ROX (PCR Biosystems). Negative control was carried out by using PCR grade water instead of cDNA template. DNA-free RNA was tested via standard PCR amplification to ensure complete removal of genomic DNA prior cDNA generation by using 16S primers (Marvasi et al. 2009). *Salmonella* genes *ygaD*, *mltB*, *srlB*, and *gutQ* were tested as genes involved in nitric oxide signaling (Ge et al. 2010), whereas *rpoD* gene was used as an internal reference gene. qPCR was performed by using the following cycles: initial denaturation at 95 °C for 2 min, 40 cycles of denaturation at 95 °C, annealing at 60 °C and extending at 65 °C for 30 s. Primers used in PCR reactions are shown in Additional file 1. Minimum requirement tests to ensure specific amplifications were performed as recommended by the MIQE Guideline (Bustin et al. 2009). PCR amplification efficiency was established by means of calibration curves. Three biological replicas and two technical replicas were done for each gene. Livak (2^−ΔΔCt^) method was used to analyse genes expression.

### Statistical analysis

The statistical software JMP (SAS) package was used to perform the one-way ANOVA analysis (p < 0.05). Tukey means separation analysis was performed in order to group the means.

## Results

### Biofilm dispersal on polypropylene and polystyrene at 4 °C

**Fig. 1:**
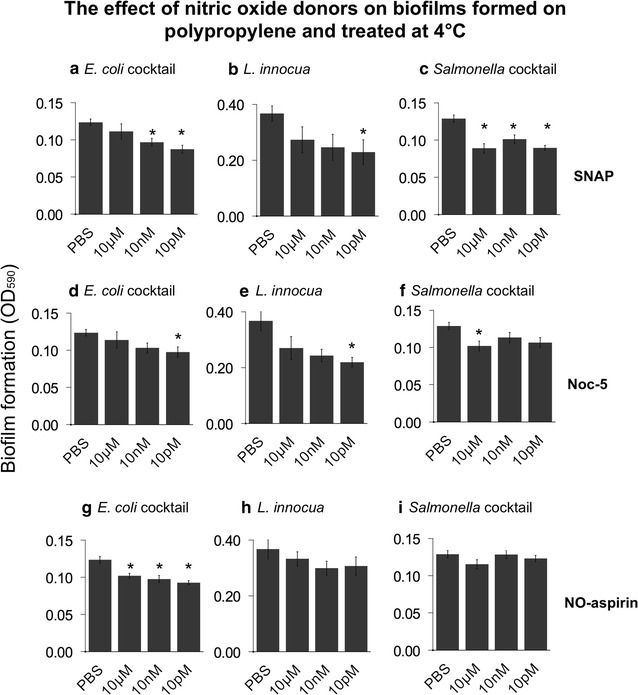
Dispersal of different preformed biofilms by the nitric oxide donors SNAP, Noc-5 and NO-aspirin on polypropylene during exposure at 4 °C. *Salmonella* and *E. coli* cocktails: see “Materials and methods” section for details about the strains. Concentrations of the nitric oxide donor are on the *x-axis*. Residual biofilms were quantified by staining with crystal violet. *Error bars* are standard errors. *Asterisk* (*) represents significant different mean when compared with the PBS treatment only (p = 0.05)

Biofilm dispersal was initially tested on polypropylene (Fig. 1). Exposure to SNAP was particularly effective in dispersing pathogenic *Salmonella*, *E. coli* and *L. innocua* biofilms which were dispersed up to 25 % when compared with the control (Fig. 1a–c). Interestingly, in the dispersal of *E. coli* we observed an inverse dose-dependent effect, already seen in our previous studies but with different donors (Marvasi et al. 2014).

When biofilms were exposed to Noc-5 the dispersal was similar as those obtained with SNAP (Fig. 1d–f). Biomass of *E. coli* cocktail, *L. innocua* and *Salmonella* cocktail were significantly reduced. In particular *L. innocua* biofilm was reduced up to 50 % when compared with the control treated with PBS only (Fig. 1e).

The treatment with NO-aspirin was not efficient as SNAP and Noc-5. Only the pathogenic *E. coli* cocktail was significantly dispersed up to 20 % when compared with the control (Fig. 1g–i).

**Fig. 2:**
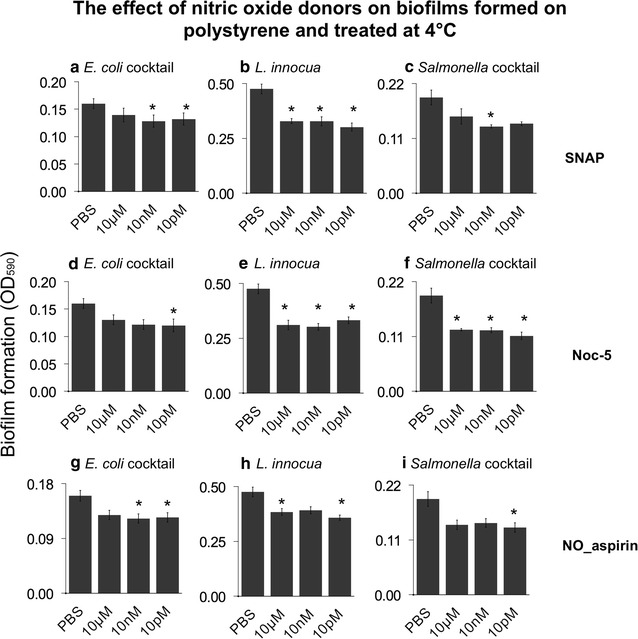
Dispersal of different preformed biofilms by the nitric oxide donors SNAP, Noc-5 and NO-aspirin on polystyrene during exposure at 4 °C. Concentrations of the nitric oxide donor are on the *x-axis*. Residual biofilms were quantified by staining with crystal violet. *Error bars* are standard errors. *Asterisk* (*) represents significant different mean when compared with the PBS treatment only (p = 0.05)

When biofilms were pre-formed on polystyrene (Fig. 2), significant dispersal was measured. SNAP treatments were effective for *E. coli*, *Listeria* and *Salmonella* cocktail, with a dispersal ranging between 15 and 20 % in all treatments (Fig. 2a–c). The treatment with Noc-5 showed significant dispersal on all the strains tested (Fig. 2d–f). The best dispersal occurred for both *L. innocua* and *Salmonella*, where significant biofilms reduction up to 30 % was measured when compared with the control.

On polystyrene, NO-aspirin was able to disperse preformed pathogenic *E. coli* cocktail biofilm up to 20 % with effective concentrations of 10 nM and 10 pM (Fig. 2g). Similarly, biofilms formed by *Listeria innocua* and *Salmonella* cocktail biofilms were dispersed by ~15 % when compared with the control (Fig. 2h, i).

### Effect of molsidomine and NO-aspirin in dispersing biofilms formed by plant pathogens

**Fig. 3:**
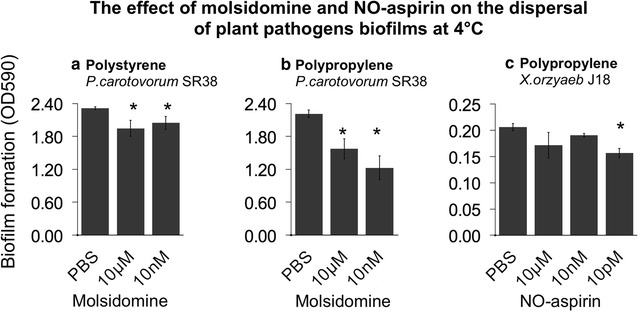
Dispersal of different preformed plant pathogens biofilms by molsidomine and NO-aspirin at 4 °C. Concentrations of the nitric oxide donors are on the *x-axis*. Residual biofilms were quantified by staining with crystal violet. *Error bars* are standard errors. *Asterisk* (*) represents significant different mean when compared with the PBS treatment only (p = 0.05)

*Pectobacterium carotovorum* SR38 and *Xanthomonas oryzae pv.oryzae* J18 biofilms were formed on polypropylene and tested with molsidomine and NO-aspirin at 4 °C (Fig. 3). Molsidomine has been chosen because previously identified as a donor with a good dispersal potential (Marvasi et al. 2014) and NO-aspirin because a potential safe molecule for application in agriculture. *P. carotovorum* SR38 biofilms were dispersed up to 10 and 30 % in polystyrene and polypropylene, respectively (Fig. 3a, b). NO-aspirin showed only a minor but significant dispersal on *X. oryzae,* up to 10 % when compared with the untreated control (Fig. 3d).

### Synergistic effect of different sanitizers with nitric oxide donors

**Fig. 4:**
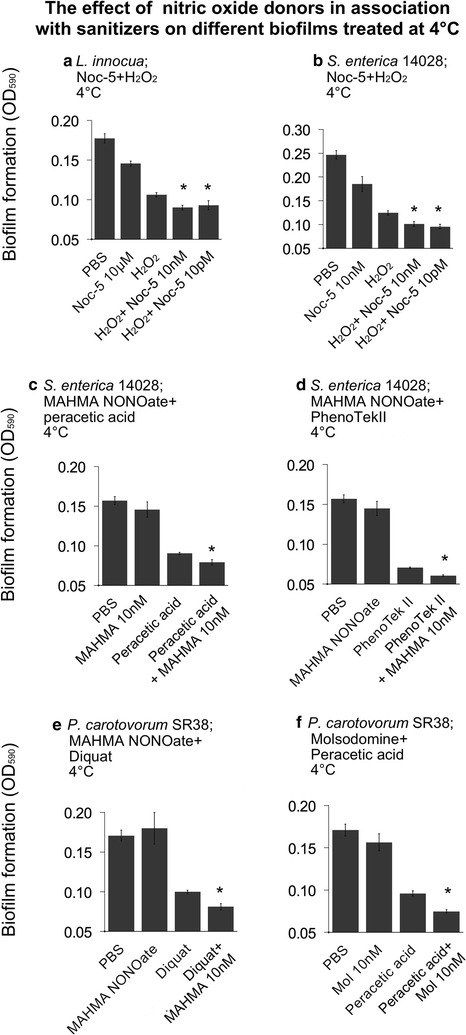
Additive effect of different sanitizers in association with nitric oxide donors. MAHMA NONOate: 6-(2-hydroxy-1-methyl-2-nitrosohydrazino)-*N*-methyl-1-hexanamine; Noc-5: 3-(aminopropyl)-1-hydroxy-3-isopropyl-2-oxo-1-triazene; NO-aspirin: 2-(acetyloxy)benzoic acid 4-(nitroxymethyl)phenyl ester. *Bars* represent the standard error. *Asterisk* (*) represents the significant synergistic effect of the nitric oxide donor in association with the sanitizer compared with the sanitizer only

For the synergistic experiments we tested the donors with best price/dispersal performance from the current and previous screenings (Marvasi et al. 2014, 2015). After revision of potential candidates we chose to test Noc-5 from the current screening, while molsidomine and MAHAMA NONOate were retrieved from previous experiments (Marvasi et al. 2014, 2015). The association of sanitizers with nitric oxide donors was tested on plant and human pathogens in order to measure to what extent synergistic effects occurred. *Listeria. innocua*, *S. enterica* and *P. carotovorum* biofilms were pre-treated with different nitric oxide donors for 6 h at 4 °C. Biofilms were then exposed to different sanitizers (Pheno-Tek II, peracetic acid 10 %, H_2_O_2_ 2 %, and Diquat) for 10 min (Fig. 4). Biofilm formed by *L. innocua* treated with Noc-5 + H_2_O_2_ showed a biofilm reduction up to 10 % when compared with H_2_O_2_ treatment alone (Fig. 4a). Significant dispersal was obtained with *S. enterica* 14028 biofilms treated with the following combinations: Noc-5 + H_2_O_2_, MAHMA NONOate + peracetic acid and MAHMA NONOate + PhenoTek II (Fig. 4b–d) showing a dispersal up to 10 % less biomass when compared with the sanitizer alone. Finally, *P. carotovorum* biofilms dispersal was limited but significant when using the algicide Diquat (widely used in agriculture) or peracetic acid (Fig. 4e, f).

### MAHMA NONOate activates the expression of Salmonella genes involved in the nitric oxide-mediated signaling

We were also interested in detecting changes in *Salmonella* gene expression during exposure to nitric oxide to confirm the fine-tuning that the donor MAHMA NONOate acts on the planktonic cells. To confirm the activation of the nitric oxide metabolic cascade upon exposure of MAHMA NONOate in *Salmonella,* relative expression of *ygaD*, *mltB*, *srlR*, and *gutQ* genes, previously identified as involved to nitric oxide signaling, was measured at 22 °C (Ge et al. 2010). All the genes tested were higher expressed in *Salmonella* cells upon exposure to 1 nM of the donor MAHMA NONOate when compared with the control. Results showed that all the genes were ~1 log_2_ more expressed than not treated cells: *ygaD* 1.68 ± 0.10, *mltB* 1.61 ± 0.10, *srlR* 1.03 ± 0.30 and *gutQ* 0.96 ± 0.10.

## Discussion

In this study we focused on the effect of off-the-shelf nitric oxide donors to disperse preformed biofilms at 37 °C and successively exposed to different donors for 6 h at 4 °C, a temperature typically used in refrigerated facilities.

The screenings presented in this work showed that the dispersals at 4 °C were moderate when compared with similar screenings carried out at higher temperatures between 22 and 25 °C (Barraud et al. 2006; Barraud et al. 2009b; Marvasi et al. 2015). The comparison with recent literature is difficult since different donors were used, however a generalized reduction of the dispersal was expected. It is well known that low temperatures may slow the nitric oxide releasing rate ultimately affecting the dispersal (Wang et al. 2005). However, beside such moderate dispersal we see potential applications in industry at low temperature. For example, in continuous flow water systems the constant application of nitric oxide donors could control biofilm formation on surfaces inaccessible for hand cleaning.

It is well known that biofilms are significantly more resistant to chlorine and other sanitizers (Corcoran et al. 2014). In this study we have shown that SNAP, Noc-5 and NO-aspirin were effective in reducing 18-h old biofilms at 4 °C (Figs. 1, 2, 3). In addition, the association of selected donors with sanitizers significantly reduced biofilms biomass in a synergistic manner (Fig. 4). Significant results are reported for the plant pathogen *P. carotovorum*, as well as for *Salmonella* and *L. innocua*. Of great interest is the dispersal of *P. carotovorum* with the algicide Diquat + MAHMA NONOate. We can speculate that constant application of such combination could be effectively used in agriculture for cleaning greenhouses or irrigation pipes.

Studies from other authors carried out at room temperature measured similar synergistic effects but wider in magnitude: A further ~80 % reduction of surface coverage of *P. aeruginosa* biofilm was measured after the association of 500 nM of sodium nitroprusside (SNP) to 10 mM H_2_O_2_ (Barraud et al. 2006, 2009b). When *Salmonella* biofilms where treated with MAHMA NONOate or molsidomine in association with the sanitizer SaniDate 12.0, biomass was reduced of an additional 20 % when compared with SaniDate 12.0 alone (Marvasi et al. 2014). Interestingly, the synergistic effect is not only limited to sanitizers but also to antibiotics and detergents. The exposure of sodium nitroprusside (500 nM) to *P. aeruginosa* greatly enhanced the efficacy of tobramycin, tetracycline and sodium dodecyl sulfate in the removal of established *P. aeruginosa* biofilms from a glass surface (Barraud et al. 2006, 2009b). Synergistic effect was also identified in the field of the chemistry of hydrogels. Encapsulation of MAHMA NONOate and molsidomine within a hydrogel composed of cellulose nanocrystals has shown a synergistic effect in dispersing *Salmonella* 1-week old biofilms (Marvasi et al. 2015).

Finally, exposure to MAHMA NONOate led to the expression of *Salmonella ygaD*, *mltB*, *srlR*, and *gutQ* genes included in the *recA*-*hydN* genomic region putatively involved in nitric oxide-mediated signaling (Marvasi et al. 2014). *mltB en*codes for membrane-bound lytic murein transglycosylase B; *ygaD* for a ribonucleoside-diphosphate reductase 2 subunit *β*; *gutQ* for an arabinose 5-phosphate isomerase; and *srlR*—glucitol operon repressor. Interestingly, relative expression of *SalmonellamltB, ygaD, gutQ* and *srlR* also increased upon infection of macrophages with *Salmonella* (Ge et al. 2010). Sustained production of nitric oxide endows macrophages with cytostatic or cytotoxic activity against bacteria (MacMicking et al. 1997). According with this result, we speculate that data from recent literature indicate that such genes may play a central role in nitric oxide detoxification, survival and replication of *Salmonella* upon exposure to nitric oxide.

Enrichment of sanitizers with nitric oxide donors could improve produce safety by expanding the tool-kit of pro-active practices for GAPs, HACCP and cleaning-in-place (CIP) protocols. However, before its application further studies must be done to: (i) Test the effectiveness of this combined products on actual industrial environment which may have multiple pathogens and very strong biofilms; (ii) To identify methods to control the nitric oxide release; (iii) To assess the neutralization/toxicity of the donors once depleted by the nitric oxide.

## Additional file

10.1186/s13568-016-0220-1 Primers used in this study.
