# Supplementary material for: Dispersal of human and plant pathogens biofilms via nitric oxide donors at 4 °C
Source: AMB Express. 2016 Jul 26;6:49. doi: 10.1186/s13568-016-0220-1 (PMC4960098; doi:10.1186/s13568-016-0220-1)
Supplement: Supplementary file 1 — 10.1186/s13568-016-0220-1 Primers used in this study. [file 13568_2016_220_MOESM1_ESM.pdf]

Supplemental Material Page

Dispersal of human and plant pathogens biofilms via nitric oxide donors at 4°C

Massimiliano Marvasi[a*], *Ian A Durie*[b], *Tania Henríquez Apablaza*[c], *Aiste Satkute*[a], *Marta Matuszewska*[a], *Raphael Carvaho Prado*[a]

Massimiliano Marvasi, Corresponding author. Department of Natural Sciences, School of Science and Technology, Middlesex University, The Burroughs, London NW4 4BT, UK. email: m.marvasi@mdx.ac.uk.

Ian A Durie, Soil and Water Science Department, University of Florida, Gainesville, FL, USA. email: durieiana@gmail.com.

Tania Henríquez Apablaza, Department of Microbiology and Mycology, University of Chile, Institute of Biomedical Sciences (ICBM). email: henriquez_apablaza@hotmail.com.

Aiste Satkute, Department of Natural Sciences, School of Science and Technology, Middlesex University, London, UK. email: aiste.satkute@gmail.com.

Marta Matuszewska, Department of Natural Sciences, School of Science and Technology, Middlesex University, London, UK. email: MM2597@live.mdx.ac.uk

Raphael Carvaho Prado, Department of Natural Sciences, School of Science and Technology, Middlesex University, London, UK. email: ralphs2@live.com

Keywords: *Salmonella enterica*, biofilms, nitric oxide donors, MAHMA NONOate, biofilm dispersal, sanitization

Supplemental Material S1. Primers used in this study.

Gene	*Forward*	*Reverse*	*Size (bps)*	*Tm °C (Fw)*	*Tm (Rev)*
ygaD	GACAAAGCCGCGTTCAAACC	GACCGTAACGACAGCAGAGT	97	60.93	59.76
mltB	GAAACCATTCGCCAACCCAG	GCAGCGTGGCGAACTATTTC	98	59.76	60.25
srlR	ACCGTGTTACAGATGGTGCC	CGTTGTCCAGTTCCGATAGC	100	60.32	58.72
gutQ	GGATGATTGAAAGCCGCGAC	GCGCGACGGATTTATCTTCC	102	59.97	59.49
*rpoD**	ACATGGGTATTCAGGTAATGGAAGA	CGGTGCTGGTGGTATTTTCA	75	59.57	58.48

* Primers designed as in Brankatschk, K., Kamber, T., Pothier, J. F., Duffy, B. and Smits, T. H. M. (2014), Transcriptional profile of *Salmonella enterica* subsp. enterica serovar Weltevreden during alfalfa sprout colonization. Microbial Biotechnology, 7: 528–544. doi:10.1111/1751-7915.12104
